# Huge Intrathoracic Lipoma Occupying the Right Hemithorax

**DOI:** 10.1002/rcr2.70479

**Published:** 2026-01-19

**Authors:** Shan Kai Ing, Jennie Geok Lim Tan, A. H. Mohd Zain, Nga Hung Ngu

**Affiliations:** ^1^ Respiratory Unit, Department of Medicine Sibu General Hospital, Ministry of Health Malaysia Sibu Sarawak Malaysia; ^2^ Department of Radiology Sibu General Hospital, Ministry of Health Malaysia Sibu Sarawak Malaysia; ^3^ Department of Pathology Sarawak General Hospital, Ministry of Health Malaysia Kuching Sarawak Malaysia

**Keywords:** computed tomography, intrathoracic lipoma, lung compression, thoracic mass

## Abstract

Intrathoracic lipomas are rare benign tumours that may attain considerable size before detection. We report a 38‐year‐old woman in whom a huge intrathoracic lipoma was incidentally identified on chest radiography during preoperative assessment. Computed tomography demonstrated a large, well‐circumscribed fat‐attenuation lesion occupying nearly the entire right hemithorax, causing near‐total lung collapse and mediastinal shift. Image‐guided biopsy confirmed a benign lipoma. This case highlights the characteristic imaging features of intrathoracic lipoma and underscores the importance of histopathological confirmation to exclude liposarcoma, even in asymptomatic patients with marked thoracic compression.

## Graphical abstract

Incidental discovery of a massive intrathoracic lipoma occupying nearly the entire right hemithorax, causing lung collapse and mediastinal shift. Computed tomography demonstrated a well‐circumscribed fat‐attenuation mass with thin septations and no solid components. Image‐guided biopsy confirmed a benign lipoma.

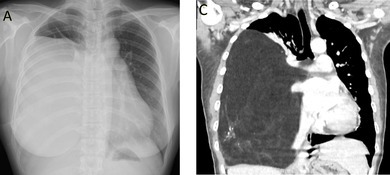

A 38‐year‐old woman with well‐controlled hypertension was referred for preoperative assessment prior to excision of a left upper‐back subcutaneous mass. Chest radiography incidentally demonstrated a large, homogeneous opacity occupying the right mid‐ and lower lung zones (Figure 1A). She was asymptomatic with normal oxygen saturation, but physical examination revealed markedly reduced breath sounds over the right hemithorax. A soft, painless, rounded subcutaneous mass was noted over the left upper back. Contrast‐enhanced computed tomography showed a huge, well‐circumscribed fat‐attenuation lesion (mean attenuation −108 Hounsfield units) measuring 14.0 × 13.0 × 23.4 cm, occupying nearly the entire right hemithorax (Figure 1B). The lesion contained thin septations and focal calcifications, without non‐adipose soft‐tissue or cystic components. There was near‐complete collapse of the right lung with mediastinal shift and diaphragmatic depression, but no evidence of invasion or extrathoracic extension (Figure 1C). Ultrasound‐guided biopsy was performed (Figure 1D) and histopathological examination confirmed a benign lipoma (Figure 1E). Intrathoracic lipomas are rare, slow‐growing benign tumours arising from mediastinal or parietal pleural fat, often discovered incidentally or when mass effect leads to compressive symptoms [1]. Characteristic imaging features include homogeneous fat attenuation with thin septations and absence of non‐adipose nodules; however, histopathological confirmation is essential to exclude well‐differentiated liposarcoma [1, 2]. Complete surgical excision is curative, with recurrence being uncommon [1, 2]. The patient declined surgical intervention and remains clinically well on follow‐up.

**FIGURE 1:**
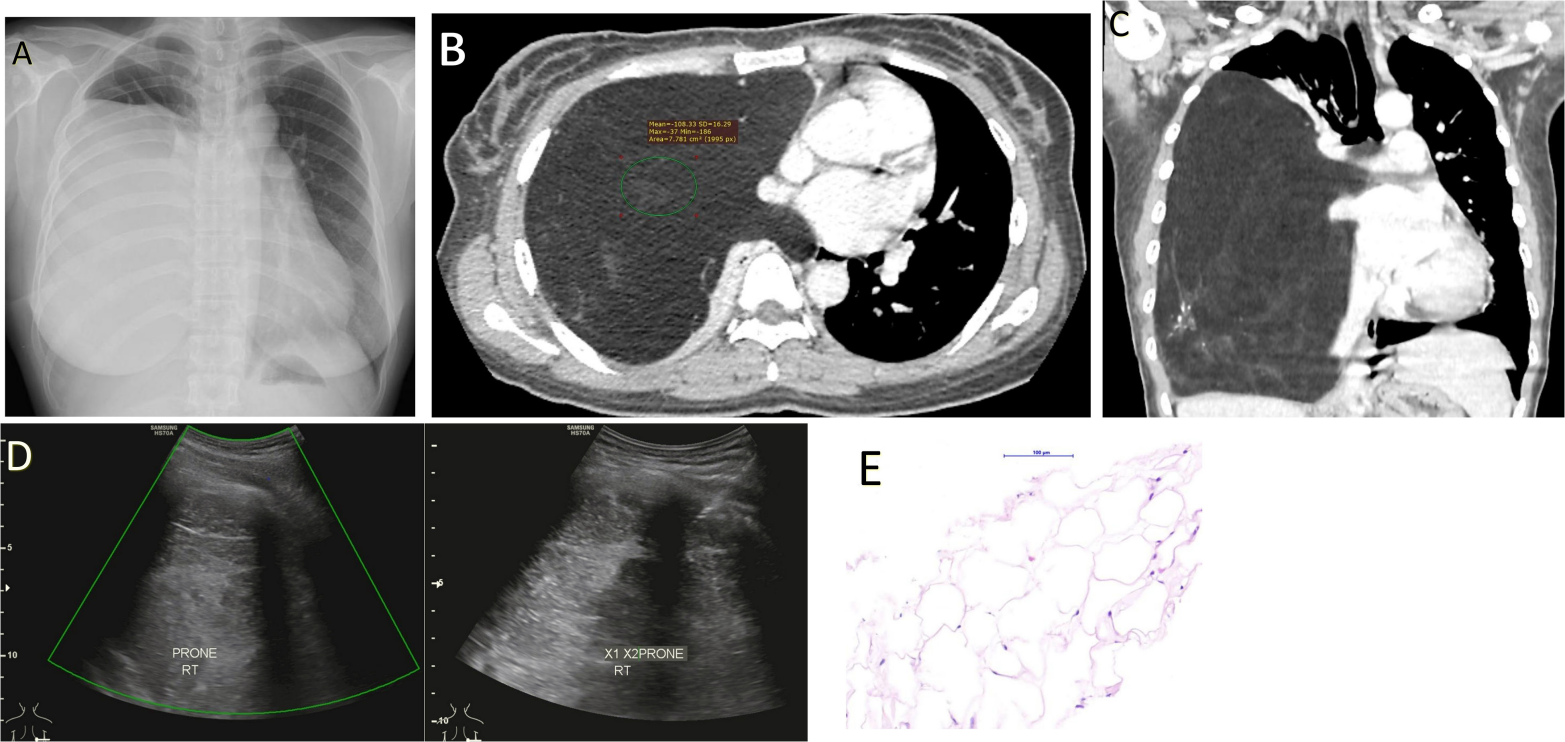
(Panel A) Chest radiograph showing huge homogeneous opacity occupying most of the right hemithorax. (Panel B) Axial contrast‐enhanced computed tomography image demonstrating a large, well‐circumscribed fat‐attenuation mass (mean attenuation −108 Hounsfield units) occupying the right hemithorax, resulting in marked compression of the right lung and contralateral mediastinal shift. (Panel C) Coronal contrast‐enhanced computed tomography image demonstrating a large right intrathoracic fat‐containing mass with thin internal septations and focal calcifications, without enhancing solid components. The lesion exerts significant mass effect, causing contralateral tracheal and mediastinal shift, as well as inferior displacement of the right hemidiaphragm and liver. (Panel D) Ultrasound image demonstrating a fat‐containing right intrathoracic mass without internal vascularity on Doppler assessment. Ultrasound‐guided biopsy was performed in the prone position, with the biopsy needle introduced through the right posterior intercostal space. (Panel E) Low‐power photomicrograph (haematoxylin and eosin stain, ×5) showing mature adipocytic tissue arranged in strips, without cytological atypia or lipoblasts, consistent with a benign lipoma.

## Author Contributions

S.K.I. conceived the case report and drafted the manuscript with contributions from J.G.L.T., A.H.M.Z. and N.H.N. N.H.N. was the managing pulmonologist, J.G.L.T. the managing radiologist, and A.H.M.Z. the managing pathologist. All authors reviewed and approved the final version of the manuscript.

## Funding

The authors have nothing to report.

## Consent

The authors declare that written informed consent was obtained for the publication of this manuscript and accompanying images using the form provided by the Journal.

## Conflicts of Interest

The authors declare no conflicts of interest.

## Data Availability

Data sharing not applicable to this article as no datasets were generated or analysed during the current study.
